# Progress in Medicine

**Published:** 1899-10-28

**Authors:** 


### Progress in Medicine.

### DISEASES OF THE RESPIRATORY TRACT.

**Phthisis.**—During the past summer the subject of tuberculosis, its ætiology, prevention, and cure, has commanded a very large amount of attention. Amongst the many meetings and conferences at which this matter has been discussed we may mention the International Congress at Berlin, the meetings of the British Medical Association, Sanitary Institute, Royal Institute of Public Health, and the general meeting of the newly-formed Association for the Prevention of Consumption. Space will not permit of our dealing at length with the proceedings of the Berlin Congress,[1] at which the study of sanatorium treatment was the prominent feature. In Germany every worker is insured against sickness, and for their own protection the assurance societies have founded sanatoria for the treatment of consumptives. Visits were paid to these sanatoria, which were found in excellent working order. The average cost per head is three marks a day. In the course of the sectional discussions many interesting statements were made by the leading authorities on tubercular diseases. Köhler dealt chiefly with the spread of the disease. It was least prevalent in England, Belgium, and Italy, and most prevalent in Hungary, Austria, and Russia. Bollinger pointed out that the frequency of swine tuberculosis was a measure of the danger from milk. Fraenkel, whilst maintaining that the breath of a phthisical person may contaminate the air, does not believe in the ubiquity of the tubercle bacillus. Pfeiffer regarded the fever of phthisis as due to secondary streptococcus infection. Courment gave a demonstration of the agglutination of tubercle bacilli by the blood of phthisical persons. Hess had succeeded in growing the tubercle bacillus in a few days in a medium consisting of agar and nutrient hyden (a soluble albumen). Virchow stated that the spread of tuberculosis from fowl to the human subject had not been proved. He rejected the doctrine of congenital and inherited tuberculosis. At the Portsmouth meeting of the British Medical Association[2] two sectional meetings discussed the subject of phthisis, the one dealing with the personal communication of tuberculosis, and the other with the preventive and remedial treatment. With regard to the first question, Groves did not advocate the active interference of sanitary authorities, but thought that if these bodies were to pay more attention to light and ventilation an improvement in the phthisis rate would result. Newsholme described the system of paid, voluntary, modified notification existing in Brighton. By this system precautionary measures could be advised and applied, and disinfection ensured. Drs. Seaton and Sidney Davies agreed. In the discussion on preventive and remedial treatment, Clifford Allbutt said that in the early stage 80 to 90 per cent. of the cases were curable, but when physical signs became obvious the proportion of cures dropped by 50 per cent. He did not regard the tubercle bacillus as the sole or even the immediate cause of the disease, but laid more stress on proclivity of tissue and individual. There were cases of phthisis in which no bacilli could be demonstrated. Phthisis generally began in the apical portion of the lung, because this was a "dead end," past which the current of air rushed, but did not enter, and thus dust accumulated. The first stage of consumption was a tuberculous endobronchitis, the earliest signs of which were localised "weak breathing," combined with patches of harsh breathing. Perhaps the best sign of early disease was to be sought in the temperature, and this should be taken every two hours. The phenomenon of clumping was too complicated and uncertain to be relied upon. Broadbent found the earliest clinical evidence of phthisis in the temperature, in the presence of myoidema, and of an occasional single sibilant râle at the apex. Douglas Powell placed great reliance on finding the bacilli. Examinations should be repeated 20 or 30 times if necessary. Beevor regarded the prevention of overcrowding, especially in poor neighbourhoods, as the most valued check to the spread of consumption. The careful measurement of the diameters of the growing chest as guide to the pretuberculous condition was emphasised by Hutchinson. The most suspicious type was the rounded chest. The part played by food, housing, heredity, and employment were touched upon by other speakers. Squire,[3] in an address on the prevention of consumption, mentions several new points. Cats, although not commonly affected, may in a household become fruitful sources of infection. A tuberculous mother may infect her child by suckling. Congenital tuberculosis is rare; direct inoculation by cuts more common. Chronically inflamed and enlarged tonsils may lead to infection of the cervical glands or to meningitis. There can be no doubt but that a predisposition to phthisis is inherited. Children with this predisposition should be brought up in the best hygienic surroundings, the nursery be sunny and well ventilated, the diet nutritious and ample. At school the hours of work and exercise carefully adjusted, and, later, care be exercised in the selection of occupation and residence. Measles, influenza, hysteria, anæmia, worry, and overwork all predispose to phthisis.

The dissemination of tuberculosis by meat[4] cannot be an important factor in the spread of the disease, for whilst the consumption per head of meat has very largely increased during the last 50 years, the tuberculosis death-rate has markedly diminished. The death-rate from tabes mesenterica, however, has shown no falling off, and this is probably to be accounted for on the ground of largely increased consumption of milk. Realising this danger in America, fourteen States have adopted and put into force regulations for checking the spread of tuberculosis by milk. In this connection we recall the advice of Thorne Thorne in the Harben lectures, namely, that all hospitals and public institutions should either have clauses in their milk contracts guarding against the possibility of tuberculous infection, or, failing this, should erect effective pasteurising plant. Wise[5] reports 10 cases in which tubercular infection was probably spread by birds. Cage-birds are especially liable to this disease, and amongst varieties mentioned were canaries and parrots; fowls, pheasants, pigeons, and poultry may also be phthisical. The dried excretions and dust from skin were the principal means of spreading the bacilli.

**Symptomology.**—Murat[6] states that an early diagnostic sign of phthisis is a sensible vibration in the affected apex on loud speaking and singing. This is brought out by making the patient make deep local expirations, and noticing the difference between the two sides. Before the appearance of physical signs the most reliable symptoms of phthisis are, according to Crookshank,[7] dyspepsia, loss of fat, night sweats, anæmia, hæmoptysis, and irregular evening rises of temperature. Consumptives are frequently found to have long, fine, silky hairs in the vertebral groove. The right upper lobe is most frequently the starting point of the disease at a point 1¼ inches below the extreme apex nearer the posterior and external borders than the internal. Hence physical signs are first to be sought for in the supra-spinous fossæ, then in the supra-clavicular fossæ, and then in the upper part of the mid axilla. The earliest change in the percussion note is a rise in pitch and a shortening of duration, and this is accompanied by a prolongation of the expiratory murmur which is separated by an interval from the inspiratory. The adventitious sound first heard is a consonating râle during inspiration after cough. An increase upwards of the area of cardiac dulness and a systolic murmur in the second left interspace during expiration are valuable early signs of phthisis. With regard to microscopic methods of examination, the detection of elastic fibres after boiling the sputum with caustic potash is diagnostic, as also is the finding of tubercle bacilli. A negative result as regards tubercle bacilli is, however, valueless unless the sputum has been examined after incubation and experimental inoculations made. Fernet[8] describes a special combination of signs found at the onset of phthisis. These signs depend on the fact that very early in the disease the tracheal and bronchial glands become enlarged and swollen. Thus, in addition to the signs at the apex of the lung, there are others at the base of the lung and the interscapular space. The enlarged bronchial glands give rise to dulness and resistance to the finger in the interscapular space on the side of the diseased lung at the level of the upper dorsal vertebræ; on auscultation hollow breath sounds are heard, the expiratory sound being the louder and longer. At the same time, there is often axillary adenopathy on the diseased side. The enlarged glands at the root of the lung press upon the lymphatic vessels and give rise to an engorgement of the lower part of the lung, which is evidenced by diminution of percussion, resonance, and subcrepitant râles. Williams[9] advocates the use of the fluorescent screen in cases of suspected phthisis. He maintains that by this method the disease may be diagnosed before physical signs are evident. On the screen the diseased portion appears darker than in health, and the excursion of the diaphragm in quiet breathing and in full inspiration is restricted on the lower side—that is, it is higher up in the chest. Walsham also speaks highly of this method of examination, and gives two skiagrams illustrating the progress of the disease in the lung.

[1] Med. Chron., June, 1899. [2] Brit. Med. Jour., Aug., 1899. [3] Lancet, July 22, 1899. [4] Med. Rec., Aug. 12, 1899. [5] Lancet, May 20, 1899. [6] Gaz. Nebd. de Med. et Chi., Mar. 5, 1899. [7] The Clinical Jour., Aug. 23, 1899. [8] Lancet, Aug. 12, 1899. [9] Amer. Jour. Med. Sci., June, 1899. [10] Lancet, July 15, 1899.

